# Ultrasonic flow ratio measured immediately after successful rotational atherectomy with stent implantation predicts major adverse cardiovascular events

**DOI:** 10.3389/fcvm.2025.1418587

**Published:** 2025-05-07

**Authors:** Tao Zhao, Qing Jin, Xi Zhang, Jiaji He, Guiping He, Qiu Chen, Yikang Sun, Pin Gan, Jilei Zhang, Xuefeng Guang, Qiang Xue

**Affiliations:** ^1^ Department of Cardiology, Yan'an Hospital of Kunming City, Yan'an Hospital Affiliated to Kunming Medical University, Kunming, China; ^2^ Kunming Cardiovascular Interventional Imaging Institute, Yan'an Hospital of Kunming City, Yan'an Hospital Affiliated to Kunming Medical University, Kunming, China; ^3^ Yunnan Key Laboratory of Cardiovascular Disease, Yan'an Hospital of Kunming City, Yan'an Hospital Affiliated to Kunming Medical University, Kunming, China

**Keywords:** rotational atherectomy, calcific coronary lesion, quantitative flow ratio, ultrasonic flow ratio, major adverse coronary events

## Abstract

**Introduction:**

The potential role of post-percutaneous coronary intervention (PCI) quantitative flow ratio (QFR) and ultrasonic flow ratio (UFR) in predicting adverse outcomes in patients with successful rotational atherectomy (RA) and stent placement remains to be defined.

**Methods:**

A total of 68 patients with highly calcific lesions, who underwent both QFR and UFR measurements after PCI with both RA and stenting, were enrolled. The major adverse coronary events (MACE) of 62 patients who completed 12-month follow-up were analyzed. The clinical characteristics of 9 patients with MACE and 53 non-MACE patients were compared. The predictors of MACE were analyzed using LASSO regression combined with Cox regression analyses.

**Results:**

Patients with MACE had more lipid-rich and mixed plaques, less stent expansion and symmetry index, and lower post-PCI QFR and UFR compared with non-MACE patients. Cox regression analyses found that patients with lower post-PCI QFR (*P* < 0.05) or lower post-PCI UFR (*P* < 0.01) had a significantly higher risk of MACE. Lasso regression was performed to select the most important predictors, and the subsequent Cox multivariate regression analyses showed that post-PCI UFR, mixed plaque, and stent expansion index were independent predictors of MACE (all *P* < 0.05). Multivariate linear regression analyses also found that changes in UFR (*P* < 0.05) and post-PCI UFR at minimal stent area (*P* < 0.01) were significantly associated with post-PCI UFR results.

**Conclusion:**

Lower value of post-PCI UFR is an independent predictor of 12-month MACE after PCI with RA and stent implantation in patients with highly calcified lesions.

## 1 Introduction

The prevalence of coronary artery calcification is increasing with accumulation of cardiovascular risk factors and population aging (1). Coronary artery calcification remains a significant challenge to successful percutaneous coronary intervention (PCI) as it is more difficult to deliver stent and achieve optimal stent expansion. Rotational atherectomy (RA) was invented more than 30 years ago and was initially used to reduce plaque burden during the era of plain old balloon angioplasty and bare-metal stent (2). Then, RA was almost abandoned because it failed to improve major adverse coronary events (MACE) and target lesion revascularization during long-term follow-up (3). In the era of second-generation drug-eluting stents, adjunct RA has been used as a plaque modification technique in severely calcified lesions to facilitate balloon dilation and stent placement (4, 5). Clinical trials demonstrated that the use of adjunct high-speed RA yielded higher procedural success rate of PCI than standard balloon pre-dilatation in treating patients with heavily calcified lesions (6–8). However, it is controversial whether RA could decrease the development of MACE during follow-up, and the incidence rate of 12-month MACE following RA and stent placement can be more than 15% in average or even higher than 20% in some studies as reported (8–10). The mechanism of high MACE incidence with RA-assisted PCI is still not fully understood, but it may be due to the fact that sicker and at high-risk patients are treated with RA. The present study aimed to investigate potential predictors of 12-month MACE in patients who underwent successful RA and stent placement.

Coronary angiography (CAG) has limited ability in evaluating the results of PCI. Post-PCI physiology studies, i.e., the measurement of fractional flow reserve (FFR), have shown that around 20% of treated vessels had suboptimal physiology after angiographically successful PCI, which highlights the importance of coronary artery physiology assessments (11–13). Importantly, high post-PCI FFR values were associated with a reduced rate of MACE and a better clinical outcome after stent placement compared with low post-PCI FFR (14). FFR has been under-utilized because of the additional time and costs associated with the use of a pressure wire (15). Recently, quantitative flow ratio (QFR) which can be quickly computed based on CAG provides an accurate and useful alternative to FFR (16). Similar to the value of FFR, studies showed that lower values of QFR after successful stent placement also predict the development of MACE (17). However, the predictive value of post-PCI FFR or QFR in patients with RA remains elusive. Intravascular ultrasound (IVUS) image study is not required but highly recommended for anatomic and physiologic assessments before and after RA and PCI especially in highly calcified lesions (18, 19). Ultrasonic flow ratio (UFR) is a novel IVUS-derived modality which estimates FFR without pressure wire and adenosine. UFR not only provides an accurate anatomic assessment of stent dimensions but also evaluates coronary artery physiology (20, 21). Studies have shown that UFR is highly concordance with FFR in assessment of coronary artery stenosis and can integrate intravascular imaging and physiological assessment in clinical practice (22). However, the prognostic value of either QFR or UFR in patients who underwent RA and stent placement was unknown. The present study was designed to test the performance of post-PCI QFR and post-PCI UFR in predicting the development of 12-month MACE after successful RA and stent placement in patients with highly calcified plaques.

## 2 Methods

### 2.1 Study population

This was a single-center, retrospective, and observational study. A total of 68 patients with coronary artery atherosclerotic and calcific lesions, who underwent both QFR and UFR measurements immediately after successful PCI including both RA and stent placement at Yanan Hospital Affiliated to Kunming Medical University, were enrolled in this study from January 2019 to January 2023. The inclusion criteria were as follows: (1) patients were greater than 18 years old; (2) diagnosed atherosclerotic coronary artery disease with severely calcified *de novo* coronary lesions using both CAG and IVUS studies; (3) underwent successful PCI with both RA and stent placement; (4) and QFR and UFR were successfully measured after RA and stenting. The exclusion criteria were as follows: (1) chronic occlusive lesions that guidewires cannot pass through; (2) in-stent restenosis or graft stenosis; (3) without stent implantation following RA; (4) use of drug-coated balloon. All data, including demographic characteristics, clinical manifestations, laboratory tests, imaging studies, procedural features, and follow-up of outcomes, were collected from the electronic medical records. The study was conducted in accordance with the ethical principles of the Declaration of Helsinki. This study was approved by the institutional review board, and written informed consent was waived by the institutional review board because of the retrospective design of the study.

### 2.2 RA and stent placement procedures

All PCI procedures were performed by experienced and credentialed interventional cardiologists in our catheterization laboratories. All patients received pretreatment with aspirin and clopidogrel or ticagrelor. During the procedure, patients received unfractionated heparin to achieve an activated clotting time of 250–300 s. The decisions to perform RA and stent placement were made by the operator after CAG and IVUS imaging studies. RA procedures were performed based on standard recommendations using the Rotablator^TM^ rotational atherectomy system (Boston Scientific, Marlborough, MA, USA) (18, 19). The burr size we selected was 1.25, 1.50, 1.75, or 2.0 mm. During RA, the burr catheter was irrigated with a cocktail flush fluid to avoid slow flow phenomenon. Completion of RA was defined as full debulking of the target lesion without premature termination of RA before proceeding to subsequent treatment. After RA, patients received a single or multiple drug-eluting stents (XIENCE PRIME stent system, Abbott Vascular, Santa Clara, CA, USA; or Endeavor Resolute stent system, Medtronic, Minneapolis, MN, USA). Then, post-dilatation with a noncompliant balloon was applied to achieve optimal angiographic and ultrasonic results with minimal residual stenosis. After stent implantation, dual antiplatelet therapy with aspirin and clopidogrel or ticagrelor were continued for at least 12 months.

### 2.3 Measurement of QFR and UFR

Pre- and post-PCI QFR was computed offline by an independent analyst through a commercial software package (AngioPlus, Pulse Medical Imaging Technology, Shanghai, China) based on baseline CAG and repeated angiography performed immediately after RA and stent placement, respectively. Details of the computational method and underlying principle of QFR were reported in previous studies (23). IVUS imaging studies were performed using a commercially available system (iLab, Boston Scientific, Marlborough, MA, USA). The IVUS catheter was advanced 10 mm distal to the lesion, and images were recorded while pulling back the catheter to the coronary ostium. Coronary lesions were classified as fibrous, lipid-rich, and mixed plaques, and ring and nodular calcification was identified based on IVUS images. Lesion length, plaque burden, minimal lumen area, stenosis at minimal lumen area, and calcium score were calculated. Pre- and post-PCI UFR values were calculated by other analyst blinded to QFR results using a commercial software package (IvusPlus, Pulse Medical Imaging Technology, Shanghai, China) based on baseline and post-PCI IVUS, respectively.

**Figure 1:**
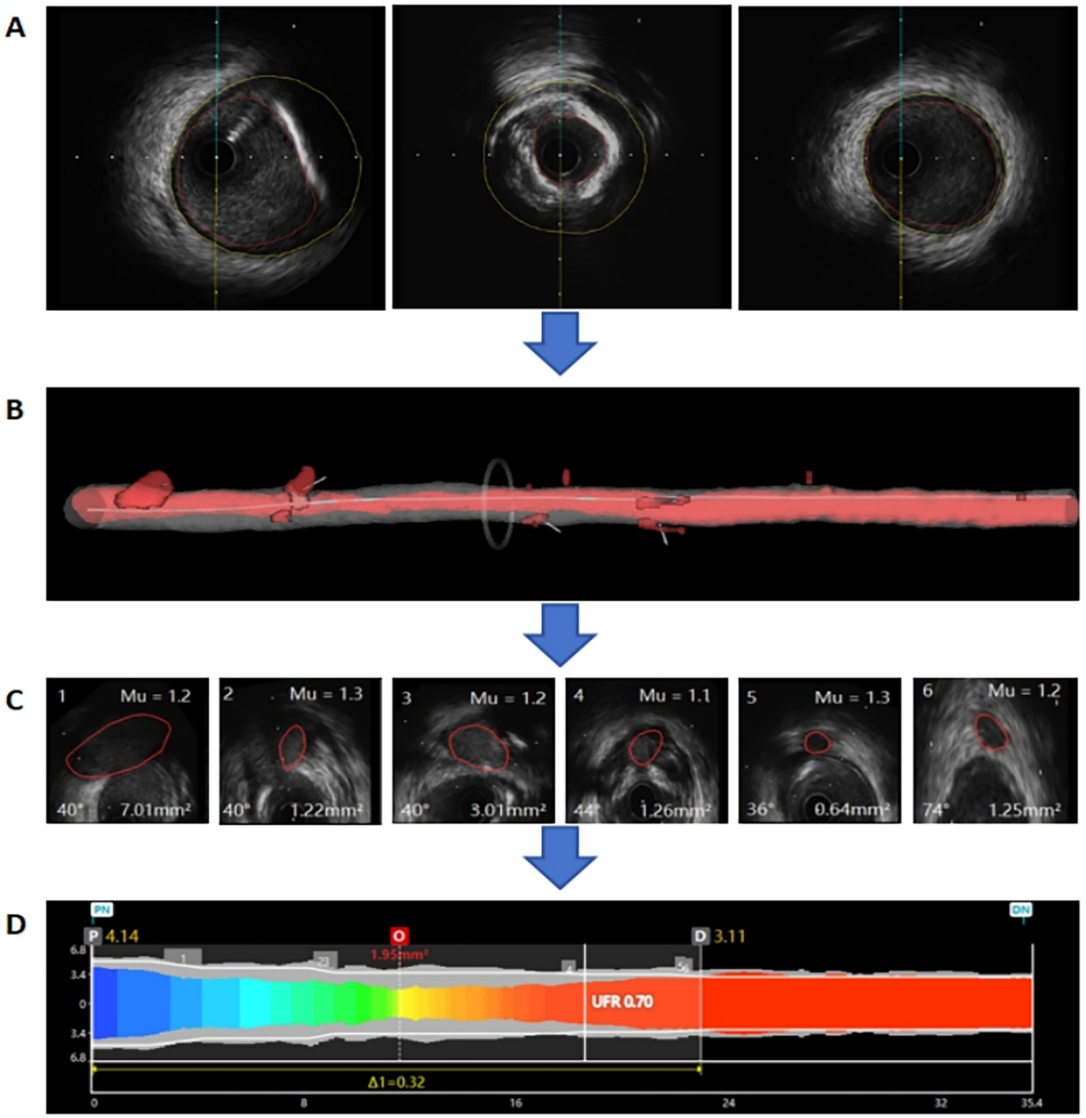
Example of the ultrasonic flow ratio (UFR) computation. **(A)** Lumen contours and external elastic lamina (EEL) were automatically delineated using a deep learning model. **(B)** The three-dimensional (3D) reconstruction of the coronary lumen was created. **(C)** The reference lumen area was derived. **(D)** The UFR pullback was obtained.

For UFR computation, firstly, lumen contours and external elastic lamina (EEL) were automatically delineated using a deep learning model on IVUS pullback images. Manual editing was allowed to correct any errors if the automatic delineated contours did not follow the lumen and EEL borders (Figure 1A). Then, a three-dimensional (3D) reconstruction of the coronary lumen was created (Figure 1B). Additionally, the software automatically reconstructed and quantified the ostia of side branches perpendicular to side branches centerlines. According to the bifurcation fractal laws, the reference lumen area was derived (Figure 1C). Then, the reference lumen area was multiplied by a fixed flow rate of 0.35 m/s to estimate the downstream perfusion flow. Finally, with a validated computational FFR method based on fluid dynamic equations, the pressure drop at each cross-section along the pullback was calculated and the UFR pullback was obtained (Figure 1D).

### 2.4 Follow-up of adverse outcomes

Follow-up was performed at 1 month, 3 months, 6 months, and 12 months after the procedure. The development of MACE within 12 months after PCI was recorded. MACE outcomes were defined as a composite of all-cause mortality, nonfatal myocardial infarction, nonfatal ischemic stroke, transient ischemic attack, stent thrombosis, and target vessel revascularization. The diagnosis of the MACE components was in accordance with the proposed deﬁnitions of 2014 ACC/AHA Key Data Elements and Definitions for Cardiovascular Endpoint Events in Clinical Trials (24).

### 2.5 Statistical analyses

All continuous variables were presented as mean ± standard error, and categorical variables were presented as frequency. Unpaired *t* test or nonparametric Mann–Whitney *U* test was used for comparisons of continuous variables, and categorical variables were compared using Chi-square test or Fisher exact test, as appropriate. To analyze predictors of MACE, we first utilized univariate Cox proportional hazard regression to evaluate the performance of post-PCI QFR and post-PCI UFR in predicting 12-month MACE. Harrell's C index was calculated and compared to evaluate the predictive performance of post-PCI QFR and post-PCI UFR. Then, we performed LASSO regression analysis to shrink potential risk factors and to preliminarily select the strongest predictors of MACE. Subsequent univariate and multivariate Cox proportional hazard regression analyses were performed on the strongest risk factors selected by the LASSO regression analysis. univariate and multivariate linear regression analyses were performed on independent predictors of post-PCI QFR and UFR. All tests were two-tailed, and statistical significance was determined at *P* < 0.05. All statistical analyses were performed using R software 4.0.2 (R Foundation for Statistical Computing, Vienna, Austria) or SPSS 22.0 (IBM, Armonk, New York, USA).

## 3 Result

### 3.1 Patient characteristics

From January 2019 to January 2023, 68 patients met the inclusion and exclusion criteria and had both QFR and UFR measured immediately after successfully RA and stent implantation. With 6 patients lost to follow-up in12 months, the adverse outcomes of 62 patients were analyzed. 9 patients developed MACE within a follow-up of 12 months. We compared the baseline features of MACE and non-MACE patients and found that the demographic characteristics, cardiovascular risk factors, medical history, and clinical manifestation were comparable between MACE and non-MACE patients (Table 1).

**Table 1:** Baseline demographic and clinical characteristics.

Variables	MACE (*n* = 9)	Non-MACE (*n* = 53)	*P*
Demographics parameters			
Age, years	69.7 ± 8.0	66.1 ± 8.6	0.24
Males, *n* (%)	3 (33.3)	31 (58.5)	0.30
BMI, kg/m^2^	23.6 ± 2.6	23.9 ± 2.2	0.72
Cardiovascular risk factors			
Hypertension, *n* (%)	6 (66.7)	38 (71.7)	0.76
Diabetes, *n* (%)	6 (66.7)	26 (49.1)	0.54
Dyslipidemia, *n* (%)	6 (66.7)	30 (56.6)	0.84
Smoking, *n* (%)	3 (33.3)	31 (58.5)	0.30
Medical history			
CHF, *n* (%)	5 (55.6)	19 (35.8)	0.45
CKD, *n* (%)	0 (0.0)	3 (5.7)	0.46
CVA, *n* (%)	2 (22.2)	11 (20.7)	0.92
Prior MI, *n* (%)	1 (11.1)	7 (13.2)	0.86
PCI, *n* (%)	1 (11.1)	16 (30.2)	0.43
Clinical presentation			
SIHD, *n* (%)	4 (44.4)	19 (35.9)	0.62
UA, *n* (%)	4 (44.4)	30 (56.6)	0.50
NSTEMI, *n* (%)	1 (11.1)	3 (5.7)	0.54
STEMI, *n* (%)	0 (0.0)	1 (1.9)	0.68

Data are expressed as mean ± SE or number of cases (frequency). BMI, body mass index; CHF, congestive heart failure; CKD, chronic kidney disease; CVA, cerebrovascular disease; PCI, percutaneous coronary intervention; MI, myocardial infarction; SIHD, stable ischemic heart disease; UA, unstable angina; NSTEMI, non-ST-elevation myocardial infarction; STEMI, ST-elevation myocardial infarction.

### 3.2 Angiographic, ultrasonic, and procedural characteristics

The results of baseline coronary angiographic analysis are shown in Table 2. Target-vessel characteristics, which included their distribution and severity of stenosis, were comparable between MACE and non-MACE patients (Table 2). The IVUS-measured minimal luminal area, lesion length, severity of stenosis, and plaque burden are similar between the two groups (Table 2). Lipid-rich plaque and mixed plaque occurred more frequently in MACE patients (both *P* < 0.01). Whereas the frequencies of ring or nodular calcification and the IVUS calcium score were similar between MACE and non-MACE patients (Table 2). Similar number of burrs per target vessel, maximal size of burrs, and size of stents were used between groups (Table 3). Patients with MACE needed two or more stents per target vessel, while nearly half of non-MACE patients received only one stent (Table 3). In addition, patients with MACE had less mean stent expansion, higher stent eccentricity index, and lower stent symmetry index (all *P* < 0.01) (Table 3). Lastly, the procedure-related complications were comparable between the two groups (Table 3).

**Table 2:** Angiographic and intravascular ultrasonic characteristics of lesions.

Variables	MACE (*n* = 9)	Non-MACE (*n* = 53)	*P*
CAG parameters			
Target vessel distribution			
LAD, *n* (%)	7 (77.8)	39 (73.6)	0.79
LCX, *n* (%)	0 (0.0)	6 (11.3)	0.29
RCA, *n* (%)	2 (22.2)	8 (15.1)	0.59
Target vessel stenosis (%)	90.0 ± 3.0	90.0 ± 8.0	0.35
Triple vessel disease, *n* (%)	8 (88.9)	42 (79.2)	0.50
Bifurcation lesion, *n* (%)	1 (11.1)	12 (22.6)	0.73
LMCA lesion, *n* (%)	7 (77.8)	23 (43.4)	0.12
IVUS parameters			
Minimal lumen area, mm^2^	2.7 ± 1.1	2.5 ± 0.7	0.91
Reference lumen area, mm^2^	11.5 ± 3.3	11.8 ± 3.2	0.78
Plaque burden at MLA, %	84.5 ± 10.0	78.1 ± 10.0	0.12
Stenosis at MLA, %	72.3 ± 14.2	70.7 ± 19.4	0.67
IVUS total lesion length, mm	63.6 ± 11.1	55.2 ± 19.2	0.21
Fibrous plaque, *n* (%)	7 (77.8)	35 (66.0)	0.76
Lipid-rich plaque, *n* (%)	9 (100.0)	22 (41.5)	<0.01
Mixed plaque, *n* (%)	8 (88.9)	16 (30.2)	<0.01
Ring calcification, *n* (%)	8 (88.9)	40 (75.5)	0.65
Nodular calcification, *n* (%)	3 (33.3)	21 (39.6)	0.72
Calcified lesion length, mm	8.5 ± 6.4	6.7 ± 5.3	0.35
Calcified lesion CSA, mm^2^	3.1 ± 1.0	3.5 ± 1.3	0.42
IVUS calcium score	2.3 ± 1.1	1.9 ± 1.1	0.27

Data are expressed as mean ± SE or number of cases (frequency). CAG, coronary angiography; LAD, left anterior descending artery; LCX, left circumflex artery; RCA, right coronary artery; LMCA, left main coronary artery; IVUS, intravascular ultrasound; MLA, minimal lumen area; CSA, cross-sectional area.

**Table 3:** Procedural characteristics of rotational atherectomy and stent placement.

Variables	MACE (*n* = 9)	Non-MACE (*n* = 53)	*P*
RA parameters			
No. of burrs per vessel			0.75
1, *n* (%)	8 (88.9)	45 (84.9)	
2, *n* (%)	1 (11.1)	8 (15.1)	
Maximal burr size, mm	1.5 ± 0.1	1.8 ± 0.3	0.13
Highest speed (×10^4^ rpm)	15.0 ± 1.0	16.0 ± 3.0	0.07
Stent parameters			
No. of stents pre vessel			0.02
1, *n* (%)	0 (0.0)	24 (45.3)	
2, *n* (%)	7 (77.8)	21 (39.6)	
3, *n* (%)	2 (22.2)	8 (15.1)	
Total stent length, mm	25.0 ± 12.0	32.0 ± 17.0	0.25
Minimal stent diameter, mm	2.7 ± 0.3	2.6 ± 0.5	0.80
Maximal stent diameter, mm	3.3 ± 0.3	3.5 ± 0.5	0.18
Minimal stent area, mm^2^	5.8 ± 1.9	6.0 ± 1.7	0.72
Mean stent expansion, %	59.7 ± 12.6	74.0 ± 13.1	<0.01
Conventional stent expansion, %	46.9 ± 6.1	50.2 ± 9.6	0.33
Stent eccentricity index	0.82 ± 0.04	0.76 ± 0.09	<0.01
Stent symmetry index	0.18 ± 0.04	0.25 ± 0.09	<0.01
Complications			
Hematoma at the puncture site, *n* (%)	2 (22.2)	13 (24.5)	0.88
Dissection, *n* (%)	3 (33.3)	7 (13.2)	0.30
Hypotension, *n* (%)	7 (77.8)	31 (58.5)	0.47
Bradycardia, *n* (%)	3 (33.3)	15 (28.3)	0.76
Chest pain, *n* (%)	0 (0.0)	5 (9.4)	0.77
QFR and UFR parameters			
Pre-PCI QFR	0.49 ± 0.15	0.52 ± 0.23	0.72
Post-PCI QFR	0.91 ± 0.03	0.94 ± 0.03	<0.05
Pre-PCI UFR	0.51 ± 0.16	0.58 ± 0.18	0.27
Post- PCI UFR	0.85 ± 0.03	0.90 ± 0.03	<0.01
Pre-PCI UFR at MLA	0.72 ± 0.27	0.74 ± 0.27	0.50
Post-PCI UFR at MSA	0.91 ± 0.04	0.92 ± 0.05	0.46
Pre-PCI UFR at calcified ring	0.78 ± 0.22	0.78 ± 0.17	0.50
Post-PCI UFR at calcified ring	0.94 ± 0.04	0.95 ± 0.04	0.54

Data are expressed as mean ± SE or number of cases (frequency). RA, rotational atherectomy; PCI, percutaneous coronary intervention; QFR, quantitative flow ratio; UFR, ultrasonic flow ratio; MLA, minimal lumen area; MSA, minimal stent area; Stent expansion index, MSA/Reference lumen area of distal stent; Conventional stent expansion index, MSA/Average of proximal and distal reference lumen area.

### 3.3 Pre- and post-PCI quantitative and ultrasonic flow ratio

The pre-PCI QFR and UFR, which were of the target vessel, at the minimal lumen area, and at calcified rings, were comparable between MACE and non-MACE patients (Table 3). Interestingly, the post-PCI QFR (0.91 ± 0.03 vs. 0.94 ± 0.03, *P* < 0.05) and post-PCI UFR (0.85 ± 0.03 vs. 0.90 ± 0.03, *P* < 0.01) measured immediately after successful RA with stent implantation were significantly lower in the MACE patients (Table 3).

### 3.4 Post-PCI QFR or UFR predicts 12-month MACE

Cox regression analyses found that patients with lower post-PCI QFR (*B*: −20.13, HR: 1.80 × 10^−9^, 95% CI: 3.80 × 10^−18^–0.86, *P* < 0.05) or lower post-PCI UFR (*B*: −42.30, HR: 4.24 × 10^−19^, 95% CI: 1.08 × 10^−30^–1.66 × 10^−7^, *P* < 0.01) had a significantly higher risk of MACE (Table 4). The accuracy of post-PCI UFR and post-PCI QFR was similar in predicting 12-month MACE (post-PCI UFR C-index: 0.82, 95% CI: 0.69–0.94 vs. post-PCI QFR C-index: 0.73, 95% CI: 0.56–0.91, *P* < 0.05) (Supplementary Table S1). Lasso regression was performed to select the most important predictor variables, and the univariate and multivariate Cox regression analyses revealed that post-PCI UFR (*P* < 0.01), mixed plaque (*P* < 0.05), and stent expansion index (*P* < 0.01) were predictors of MACE in post-PCI patients (Table 5).

**Table 4:** Predictive value of post-PCI UFR and QFR on MACE.

Variables	*B*	HR	95% CI	*P*
Post-PCI QFR	−20.13	1.80 × 10^−9^	3.80 × 10^−18^–0.86	0.04
Post-PCI UFR	−42.30	4.24 × 10^−19^	1.08 × 10^−30^–1.66 × 10^−7^	<0.01

PCI, percutaneous coronary intervention; QFR, quantitative flow ratio; UFR, ultrasonic flow ratio.

**Table 5:** Predictors of MACE following PCI.

Variables	Univariate Cox regression			Multivariate Cox regression		
	HR	95% CI	*P*	HR	95% CI	*P*
Post-PCI UFR	4.24 × 10^−19^	1.08 × 10^−30^–1.66 × 10^−7^	<0.01	3.12 × 10^−14^	7.86 × 10^−25^–0.01	0.01
Mixed plaque	14.91	1.86–119.40	0.01	10.82	1.30–89.99	0.03
Stent expansion index	0.93	0.88–0.97	<0.01	0.94	0.89–1.00	0.04

PCI, percutaneous coronary intervention; QFR, quantitative flow ratio; UFR, ultrasonic flow ratio.

### 3.5 Factors associated with post-PCI QFR and post-PCI UFR

Considering that post-PCI QFR and UFR are valuable in predicting MACE, we performed linear regression analyses to identify factors associated with these two alternative measurements of FFR. Univariate linear regression analyses revealed that distal stent length and total stent length are associated with post-PCI QFR (both *P* < 0.01), however, multivariate linear regression analyses failed to confirm them as independent predictors (Table 6). Univariate linear regression analyses found that changes in UFR, post-PCI UFR at minimal stent area, total lesion length, and number of stents used for each vessel were associated with post-UFR (*P* < 0.05 or *P* < 0.01), and multivariate linear regression analyses confirmed that changes in UFR (*P* < 0.05) and post-PCI UFR at minimal stent area (*P* < 0.01) were independent predictors of post-PCI UFR (Table 7).

**Table 6:** Factors associated with the post-PCI QFR value.

Variables	Univariate linear regression			Multivariate linear regression			
	*B*	SE	*P*	*B*	SE	*β*	*P*
Distal stent length	2 × 10^−3^	4.82 × 10^−6^	<0.01	1 × 10^−3^	1 × 10^−3^	0.32	0.09
Total stent length	1 × 10^−3^	2.72 × 10^−6^	<0.01	4.41 × 10^−6^	3.58 × 10^−6^	0.23	0.23

PCI, percutaneous coronary intervention; QFR, quantitative flow ratio.

**Table 7:** Factors associated with the post-PCI UFR value.

Variables	Univariate linear regression			Multivariate linear regression			
	*B*	SE	*P*	*B*	SE	*β*	*P*
Changes in UFR	−0.93	0.03	<0.01	−0.89	0.04	−0.92	<0.01
Post-PCI UFR at MSA	0.32	0.09	<0.01	0.06	0.03	0.09	0.04
Total lesion length	−1 × 10^−3^	2.49 × 10^−6^	0.01	−2.65 × 10^−5^	1.10 × 10^−6^	−0.01	0.81
No. of stents pre vessel	−0.02	0.01	<0.01	−1 × 10^−3^	3 × 10^−3^	−0.03	0.66

PCI, percutaneous coronary intervention; UFR, ultrasonic flow ratio; MSA, minimal stent area.

## 4 Discussion

The present study was conducted to investigate the potential role of QFR and UFR, computing immediately after successful PCI with both RA and implantation, in the prediction of adverse events within 12 months. To minimize confounding factors, we only selected patients with highly calcified lesions and undergoing successful RA and revascularization. Moreover, QFR and UFR were computed offline by two independent analysts blinded to the study. The main findings are as follows. First, patients who developed 12-month MACE had more lipid-rich and mixed plaques, less stent expansion and symmetry index, and lower post-PCI QFR and UFR compared with non-MACE patients. Second, post-PCI QFR and post-PCI UFR had excellent and similar prognostic value for MACE in univariate Cox analysis, while LASSO regression and multivariate Cox analysis identified that only post-PCI UFR, mixed plaque, and stent expansion index were independent predictors of 12-month adverse events. Third, changes in UFR and post-PCI UFR at minimal stent area independently influenced post-PCI UFR measurements.

Post-PCI FFR and QFR have been used to predict adverse events in patients underwent stent implantation, however, to the best of our knowledge the present study is the first to investigate the prognostic value of post-PCI UFR in patients underwent both RA and stent implantation. FFR as the prototype and gold standard measurement of coronary flow reserve has been utilized to assess the physiology of coronary artery disease, which is superior to CAG that only anatomically assesses stenotic lesions (25). Post-PCI FFR detects residual coronary artery disease burden after revascularization by measuring flow reduction, and the outcomes of FFR-guided PCI are superior to angiography-guided management of coronary artery disease (26, 27). Importantly, post-PCI FFR serves as a reliable independent predictor of target vessel failure and adverse events (28–30). Unfortunately, post-PCI FFR is not routinely computed in most catheterization labs in daily clinical practice partially due to extra costs associated with pressure wires, prolonged procedure duration, and potential adverse effects of adenosine administration (15). QFR and UFR are adenosine-independent pressure indexes of coronary artery stenosis and have been developed as substitutes for FFR.

### 4.1 Clinical and procedural insights into QFR

QFR is a pressure wire-free assessment of coronary physiology based on CAG without the need of epicardial vasodilation by adenosine administration. QFR has a high correlation and agreement with FFR (31) and is used to optimize PCI of multivessel disease complying with the current guidelines (32). Post-PCI QFR was associated with the development of MACE (33), and lower values of QFR after successful revascularization by stent placement predicted subsequent adverse events (17). Moreover, a previous study showed that post-PCI QFR had a high predictive value for target lesion failure during a 3-year follow-up after RA and stent placement in patients with heavily calcified lesions (34). This finding is similar to the current study which showed that post-PCI QFR was an excellent predictor of 12-month MACE after RA and stent implantation in univariate Cox regression analysis. However, post-PCI QFR as a variable was excluded by LASSO regression which we performed to shrink potential risk factors and to preliminarily select the strongest predictors of MACE. It is possible that post-PCI QFR was highly correlated with post-PCI UFR, but the prognostic value of post-PCI QFR might be not as strong as post-PCI UFR (Supplementary Table S1), so it was abandoned in LASSO regression analysis. If RA and stent placement were not guided by IVUS or UFR was not available, post-PCI QFR would still be a valuable predictor of adverse events after RA. QFR has demonstrated good inter-core laboratory reproducibility in assessing the physiological significance of coronary stenosis (35). However, operator, angiographic quality, and the coronary artery stenosis severity and imaging system factors can influence its accuracy (36). Therefore, adherence to standardized imaging protocols and operator training is essential to ensure accuracy and consistency across different operators and systems.

### 4.2 Clinical and procedural insights into UFR

UFR is a novel FFR alternative modality that can be fast computed based on IVUS imagines. IVUS has been widely used to optimize PCI especially in patients with complex and severely calcified lesions. Clinical expert consensus documents recommend using IVUS before, during, and after RA to improve procedural success and safety (18, 19, 37), and the use of IVUS for complex and calcified lesions was associated with decreased risk of target vessel revascularization and mortality (20, 38). Therefore, UFR is readily available in most cases when RA is performed to modify calcified lesions. It has been proved that UFR has a strong correlation with FFR and can be used to accurately assess hemodynamic significance of coronary artery stenosis (21), and the diagnostic performance of UFR is non-inferior to QFR (22). In previous studies, UFR were confirmed to have a better performance in left main coronary artery diseases, multivessel diseases, bifurcation lesion diseases (21, 22, 39, 40). A previous study showed that lesion-specific UFR was an independent predictor of three-year MACE after PCI with stent placement (40), but UFR was not yet used to predict adverse outcome of calcific lesions following RA. The present study provides compelling evidence that post-PCI UFR is an independent predictor of 12-month MACE in addition to mixed plaque and stent expansion index. Combination of intravascular image study and coronary physiological assessment is recommended in PCI to enable optimal revascularization. UFR is a modality that combines morphological anatomical features and functional assessment without using extra devices. Post-PCI UFR values may be used to define the endpoint of PCI in order to optimize the results of PCI in the future. In the present study, lower post-PCI UFR was associated with longer total lesion length and more stents used in each target vessel. This finding suggests that diffuse long coronary artery lesions may require more efforts to achieve optimal revascularization as this type of lesions were notoriously associated with higher incidence of MACE and target vessel revascularization (41, 42). UFR can be readily integrated into routine clinical practice as it utilizes standard IVUS imaging, requiring no additional equipment, contrast agents, or hyperemic agents.

Compared with other diagnostic modalities, such as optical coherence tomography (OCT) and its based optical flow ratio (OFR), which is another novel FFR alternative modality, UFR provides distinct advantages. Firstly, UFR enables a one-step, streamlined assessment of coronary physiology and lesion morphology without requiring additional equipment, thereby enhancing procedural efficiency and reducing costs. Secondly, a meta-analysis demonstrated that UFR has superior sensitivity and specificity in identifying hemodynamically significant coronary stenosis compared to OFR (43). Probably, because IVUS has better tissue penetration, higher chances of covering the entire lesions and wider clinical penetration than OCT (21). Future comparative studies examining UFR and OFR within the same clinical settings could further elucidate their respective advantages and limitations. Near-infrared spectroscopy-intravascular ultrasound (NIRS-IVUS), a newer but not widely available modality, integrates two imaging technologies and is primarily used for identifying high-risk plaques (44). While NIRS-IVUS excels at characterizing plaque composition and structural assessment, UFR offers additional benefits by delivering a streamlined physiological assessment of coronary stenosis without requiring specialized dual-modality equipment. As emphasized in current guidelines for coronary artery revascularization (45), integrating multiple diagnostic tools and clinical judgment is essential for optimal PCI outcomes.

### 4.3 Clinical application of UFR and QFR

**Figure 2:**
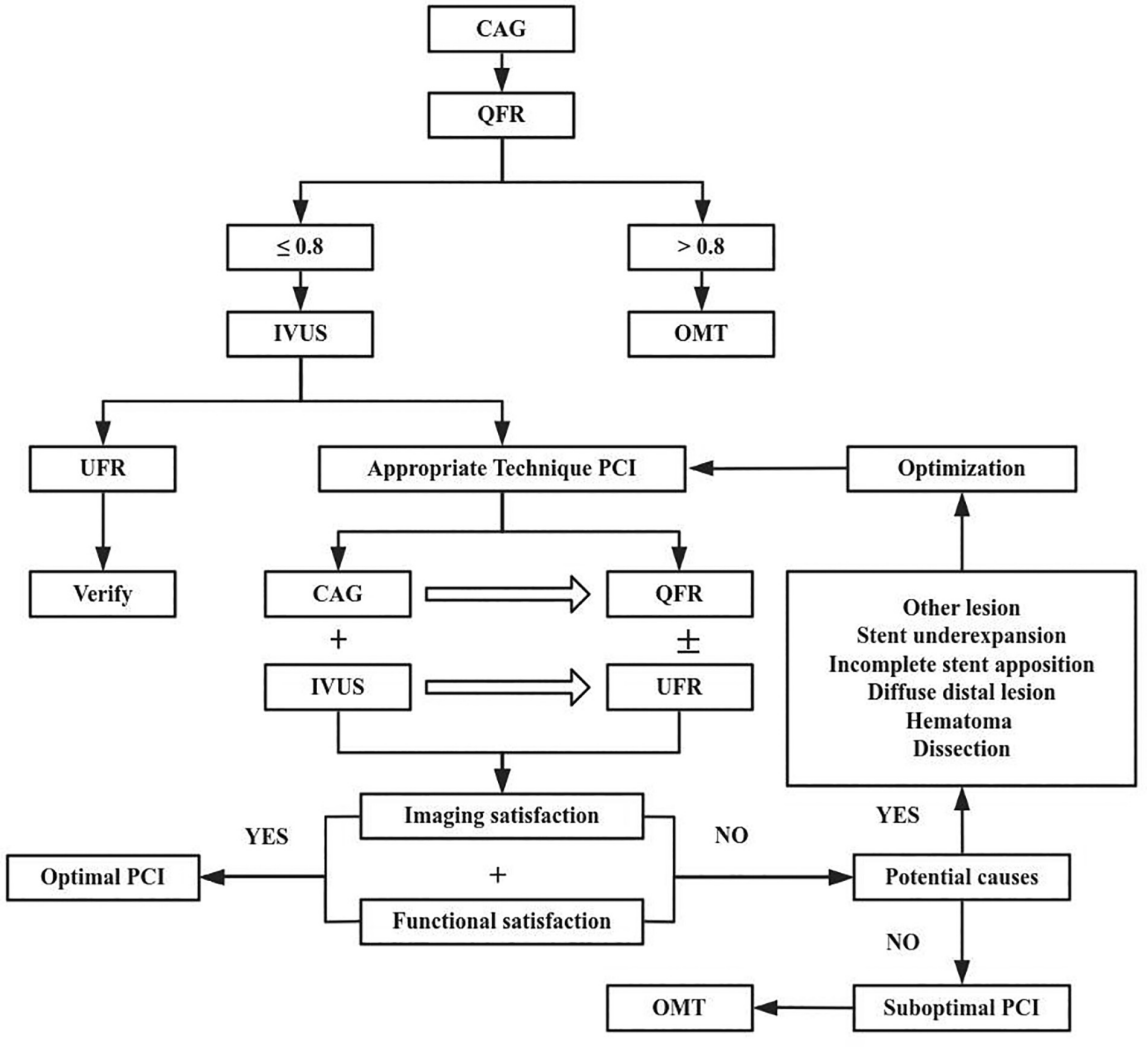
Process of optimal PCI with quantitative flow ratio (QFR) and ultrasonic flow ratio (UFR). CAG, coronary angiography; IVUS, Intravascular ultrasound; OMT, Optimal medical therapy.

In practice, UFR could be complementary to QFR (Figure 2). QFR is well suited for diagnostic procedures, whereas the UFR supports complex PCI optimization. Although UFR based on IVUS with OptiCross catheters provides key physiological insights, OptiCross catheters crossing lesions in severe stenosis cases may be challenging before procedure. Although in-procedure QFR has been validated as feasible and safe with high diagnostic accuracy, in patients with complex coronary anatomy angiographic projections without vessel overlapping or significant foreshortening might be difficult to obtain. Therefore, in order to guiding optimal PCI to imaging satisfaction and functional satisfaction, we can combine QFR with UFR to assess lesion-specific ischemia but should be complemented by anatomical imaging tools, such as IVUS to evaluate plaque morphology and vessel structure. Additionally, patient-specific factors must guide decision-making to ensure tailored treatment strategies.

RA was invented more than three decades ago, and the use of RA has waxed and waned a few times over the years (4). RA has been used to facilitate PCI and improve procedural success rates in the era of drug-eluting stents (46), while it is always questionable whether the use of RA can benefit long-term outcomes. The use of IVUS increases procedural success rate and the safety of RA (47, 48), but it is still unknow whether IVUS can improve long-term outcome after RA. It is challenging to compare the outcomes between RA combined with stenting vs. stenting alone because stent placement without RA could be impossible in certain complex and highly calcified plaques. However, it should be feasible to investigate whether the use of IVUS parameters or specifically UFR as a potential endpoint to guide revascularization can improve long-term outcome after PCI with RA.

To sum up, IVUS combined with CAG has been used to evaluate the indications and effect of RA to optimize PCI of the calcific lesions complying with the current guidelines. QFR and UFR, which are pressure wire-free assessments of coronary physiology based on CAG and IVUS, could do one-stop evaluation of structure and physiology of coronary artery lesions without extra facility. As well as, QFR and UFR may predict adverse outcome following RA. It's convenient to facilitate PCI and favorable to improve prognosis for patients with less expense. Whether both could additionally provide an alternative optimal algorithm for complex PCI in the light of the study deserves further study in the future (Figure 2).

## 5 Limitation

This study's limitations include its single-centre, retrospective design, relatively small sample size and short follow-up period, which inherently introduces potential selection bias. Coronary artery disease is multiple-etiology and chronic disease, so longer-term studies are necessary to assess the sustained efficacy and safety of the intervention, while the 12-month follow-up provides valuable insights into early outcomes. Future research with extended follow-up periods will be critical to better understand the long-term benefits and potential limitations of the approach. Then, despite the analysis being conducted by automatic artificial intelligence or two experienced operators, individual variability is inevitable in the process of TIMI frame or IVUS frame counting, thus introduces error to some degree, especially in those cases with relatively poor quality of image. Additionally, we would like to point out that the majority of our patients had acute coronary syndrome (ACS) instead of stable ischemic heart disease. The use of RA in patients with ACS is still controversial as the current revascularization guidelines and expert consensus documents only recommend applying RA to treat calcified lesions in patients with chronic coronary syndromes (18, 19, 37, 45, 49). Recent studies showed that RA is feasible in patients with ACS, resulting in comparable procedural outcomes but a higher long-term MACE rate compared to the use of RA in patients with chronic coronary syndromes. This should be considered when comparing the rate of MACE in the present study with the results of future studies.

## 6 Conclusion

Lower value of post-PCI UFR is an independent predictor of adverse events after PCI with both RA and stent implantation in patients with highly calcified coronary lesions. Post-PCI QFR may also have prognostic value if UFR is not available.

## Data Availability

The raw data supporting the conclusions of this article will be made available by the authors, without undue reservation.
