# Supplementary material for: Ultrasonic flow ratio measured immediately after successful rotational atherectomy with stent implantation predicts major adverse cardiovascular events
Source: Front Cardiovasc Med. 2025 May 7;12:1418587. doi: 10.3389/fcvm.2025.1418587 (PMC12092348; doi:10.3389/fcvm.2025.1418587)
Supplement: Supplementary file 1 [file Table1.docx]

**Table S1.** The accuracy of post-PCI UFR and post-PCI QFR in predicting 12-month MACE

| **Variables** | **C-index** | **95%CI** | ***P*** |
| --- | --- | --- | --- |
| Post-PCI UFR | 0.819 | 0.694 - 0.943 | 0.023 |
| Post-PCI QFR | 0.733 | 0.561 - 0.905 |  |

PCI, percutaneous coronary intervention; QFR, quantitative flow ratio; UFR, ultrasonic flow ratio.
